# Association between eating behavior patterns and the therapeutic efficacy of GLP-1 receptor agonists in individuals with type 2 diabetes: a multicenter prospective observational study

**DOI:** 10.3389/fcdhc.2025.1638681

**Published:** 2025-09-17

**Authors:** Yuya Koide, Takehiro Kato, Makoto Hayashi, Hisashi Daido, Takako Maruyama, Takuma Ishihara, Kayoko Nishimura, Shin Tsunekawa, Daisuke Yabe

**Affiliations:** ^1^ Department of Diabetes, Endocrinology and Metabolism, Graduate School of Medicine, Gifu University, Gifu, Japan; ^2^ Department of Rheumatology and Clinical Immunology, Graduate School of Medicine, Gifu University, Gifu, Japan; ^3^ Department of Internal Medicine, Matsunami General Hospital, Gifu, Japan; ^4^ Division of Diabetes and Endocrinology, Gifu Prefectural General Medical Center, Gifu, Japan; ^5^ Division of Diabetes and Endocrinology, Gifu Municipal Hospital, Gifu, Japan; ^6^ Innovative and Clinical Research Promotion Center, Gifu University Hospital, Gifu, Japan; ^7^ Division of Clinical Nutrition and Metabolism, Gifu University Hospital, Gifu, Japan; ^8^ Center for One Medicine Innovative Translational Research, Gifu University Institute for Advanced Studies, Gifu, Japan; ^9^ Department of Diabetes, Endocrinology and Nutrition, Kyoto University Graduate School of Medicine, Kyoto, Japan

**Keywords:** GLP-1 receptor agonists, type 2 diabetes, eating behavior, weight loss, personalized medicine

## Abstract

**Background:**

Glucagon-like peptide-1 receptor agonists (GLP-1RAs) are widely used to improve glycemic control and induce weight loss in individuals with type 2 diabetes (T2D), yet treatment responses vary significantly among individuals. Eating behavior has been hypothesized to influence therapeutic efficacy, but supporting evidence remains limited.

**Methods:**

In this multicenter, prospective observational study, we enrolled 92 individuals with T2D initiating GLP-1RA therapy (liraglutide, dulaglutide, oral semaglutide, or injectable semaglutide) at four institutions in Gifu Prefecture, Japan. Participants were assessed at baseline, 3 months, and 12 months for clinical parameters, dietary intake, and eating behaviors using validated tools (Food Frequency Questionnaire and the Japanese version of the Dutch Eating Behavior Questionnaire [DEBQ-J]). Primary and secondary outcomes included changes in HbA1c, body weight, and eating behavior patterns over 12 months.

**Results:**

GLP-1RA therapy significantly reduced HbA1c, body weight, and body fat percentage at 12 months. Notably, external eating scores showed a sustained decrease, while emotional and restrained eating scores exhibited transient changes. Higher baseline external eating scores were independently associated with greater weight reduction and showed a trend toward enhanced glycemic improvement. No significant associations were observed between emotional or restrained eating scores and clinical outcomes.

**Conclusion:**

This study demonstrates that GLP-1RAs improve both metabolic parameters and external eating behavior in T2D individuals. External eating emerged as a potential behavioral marker predictive of treatment response. These findings suggest that integrating eating behavior assessments may help personalize GLP-1RA therapy and optimize outcomes in clinical practice.

**Clinical trial registration:**

UMIN Clinical Trials identifier, UMIN000045362.

## Highlights

- GLP-1 receptor agonists (GLP-1RAs) significantly reduced HbA1c, body weight, and body fat percentage after 12 months in real clinical settings.
- GLP-1RAs consistently reduced external eating scores over time. External eating refers to eating behavior triggered by external cues such as appetizing smells, the visual appeal of food, or observing others eat.
- GLP-1RAs also led to a short-term reduction in emotional eating, which is characterized by eating in response to negative emotions such as stress, anger, sadness, or anxiety. However, this effect was not sustained in the long term.
- Higher baseline external eating scores were associated with greater weight loss and a trend toward improved glycemic outcomes.
- In contrast, emotional eating scores at baseline were not significantly associated with changes in weight or glycemic control following GLP-1RA treatment.
- External eating scores may serve as a useful behavioral predictor of treatment response to GLP-1RAs and help guide personalized therapeutic strategies in clinical practice.

## Introduction

Glucagon-like peptide-1 receptor agonists (GLP-1RAs) are well-established therapeutic agents that exert potent glucose-lowering effects by stimulating insulin secretion, suppressing glucagon secretion in a glucose-dependent manner, and delaying gastric emptying (1). Beyond their glycemic benefits, GLP-1RAs also reduce food intake by modulating appetite-regulating pathways, resulting in clinically meaningful weight loss (1, 2). These multifaceted effects have positioned GLP-1RAs as a cornerstone in the management of type 2 diabetes (T2D), particularly in individuals with obesity. Although T2D in Japan and other East Asian countries is primarily characterized by lower levels of obesity and impaired insulin secretion compared to Western populations (3), GLP-1 receptor agonists are increasingly used among younger individuals with type 2 diabetes who are overweight or obese in these regions. Despite their proven efficacy, the glycemic and weight-lowering effects of GLP-1RAs exhibit considerable interindividual variability. For example, long-acting GLP-1RAs have demonstrated glucose-lowering effects closely associated with residual pancreatic β-cell function and mass, as supported by both clinical and preclinical imaging studies (4–8). Moreover, previous reports have suggested that individuals showing a particular eating behavior may experience improved glycemic control with GLP-1RAs but often achieve less pronounced weight loss (9). However, the extent to which eating behavior influences the therapeutic response to GLP-1RAs remains unclear.

Eating behavior is a critical determinant in T2D management (10) and reflects a complex interplay of psychosocial, behavioral, and environmental factors (11). Among the key dimensions of eating behavior, external eating, emotional eating, and restrained eating have been implicated in overeating and weight management difficulties (12, 13). External eating is characterized by food consumption triggered by external stimuli, such as the sight or smell of food, and has been consistently linked to weight gain, especially in modern food-rich environments (14). Emotional eating is driven by negative emotions, such as anxiety or sadness, rather than physiological hunger and is associated with excessive intake of calorie-dense foods (15, 16). In contrast, restrained eating is defined by the conscious restriction of food intake for weight control purposes and, when practiced in moderation, is linked to improved weight management (17).

Although these eating behavior patterns are hypothesized to influence the clinical efficacy of GLP-1RA therapy, direct evidence remains limited. To address this gap, we aimed to examine the relationship between eating behavior patterns and both glycemic and weight-reducing outcomes following GLP-1RA initiation in individuals with T2D.

## Materials and methods

This multicenter, prospective observational study was conducted at four institutions in Gifu Prefecture, Japan (Gifu University Hospital, Matsunami General Hospital, Gifu Prefectural General Medical Center, and Gifu Municipal Hospital). Individuals with T2D who were deemed suitable for GLP-1RA therapy by their attending physicians between August 2021 and December 2024 were screened for eligibility. Informed consent was obtained from all participants after a full explanation of the study objectives. The study was approved by the Ethics Committee of the Gifu University Graduate School of Medicine (Approval No.: 2021-B021) and conducted in accordance with the Declaration of Helsinki. The inclusion criteria were adults with T2D deemed appropriate candidates for GLP-1RA therapy. Exclusion criteria included: (1) individuals unable to comply with study procedures; and (2) individuals judged to be unsuitable for participation by the investigators. The selection of a specific GLP-1 receptor agonist was determined by the attending physician, based on routine clinical judgment, and was not guided by any predefined criteria. Clinical and demographic data—including age, sex, BMI, duration of diabetes, smoking status, comorbidities, laboratory values, and current medications—were collected from electronic medical records. Hypertension and dyslipidemia were defined based on physician diagnosis and/or relevant pharmacotherapy. Participants were assessed at baseline (prior to GLP-1RA initiation), 3 months, and 12 months post-initiation. Assessments included laboratory testing, body composition analysis via bioelectrical impedance, and two validated questionnaires: the Food Frequency Questionnaire (FFQ) and the Japanese version of the Dutch Eating Behavior Questionnaire (DEBQ-J) (17–19). Laboratory parameters included complete blood count, fasting plasma glucose, C-peptide, HbA1c, AST, ALT, γ-GTP, amylase, lipase, uric acid, BUN, creatinine, total cholesterol, HDL-cholesterol, and triglycerides. Dietary intake was assessed using the FFQ, which estimates total energy and macronutrient intake over a 1- to 2-month period. The FFQ’s reproducibility and validity have been previously established (18, 19). Eating behavior was assessed using the DEBQ-J, consisting of 33 items categorized into external eating, emotional eating, and restrained eating subscales (11). Responses were rated on a 5-point Likert scale (1 = “never” to 5 = “very often”), with higher scores indicating stronger tendencies toward the respective eating behaviors. The DEBQ has been widely validated across diverse populations, including Japanese cohorts (20–22). The addition or reduction of anti-diabetes agents, including insulin, was determined at the discretion of the attending physicians. The primary outcome was the change in HbA1c, and secondary outcomes were 1) changes in body weight and body composition; 2) longitudinal changes in eating behavior scores; 3) interactions of changes in HbA1c after 12-month GLP-1RA therapy with eating behavior scores and GLP-1RA agents; 4) interactions of changes in bodyweight after 12-month GLP-1RA therapy with eating behavior scores and GLP-1RA agents. As an exploratory analysis, we also evaluated changes in total energy intake and macronutrient intake.

Clinical characteristics were summarized as mean and SD for continuous variables and frequencies for categorical variables. HbA1c, body weight, and body fat percentage at 12 months after GLP-1RAs and at baseline were compared using the paired-t test. We used a multivariable regression model with HbA1c and weight change over 12 months after GLP-1RA therapy as the dependent variables to evaluate the impact of the three DEBQ scores. Covariates included baseline HbA1c, baseline BMI, duration of diabetes, and GLP-1RA (liraglutide, dulaglutide, oral semaglutide, injectable semaglutide). Missing values were imputed using additive regression. The factors included in the additive regression were the same as those included in the multivariable linear regression model, and the number of imputations was set to 5. Specifically, the aregImpute function in the “Hmisc” package of R was used. The comparisons of DEBQ scores between measurement points were conducted using the paired t-test. The two-sided significance level was set at 5%. A two-sided p <0.05 were considered statistically significant. All analyses were performed using R version 4.4.1 (The R Project for Statistical Computing, Vienna, Austria, www.r-project.org).

## Results

**Figure 1:**
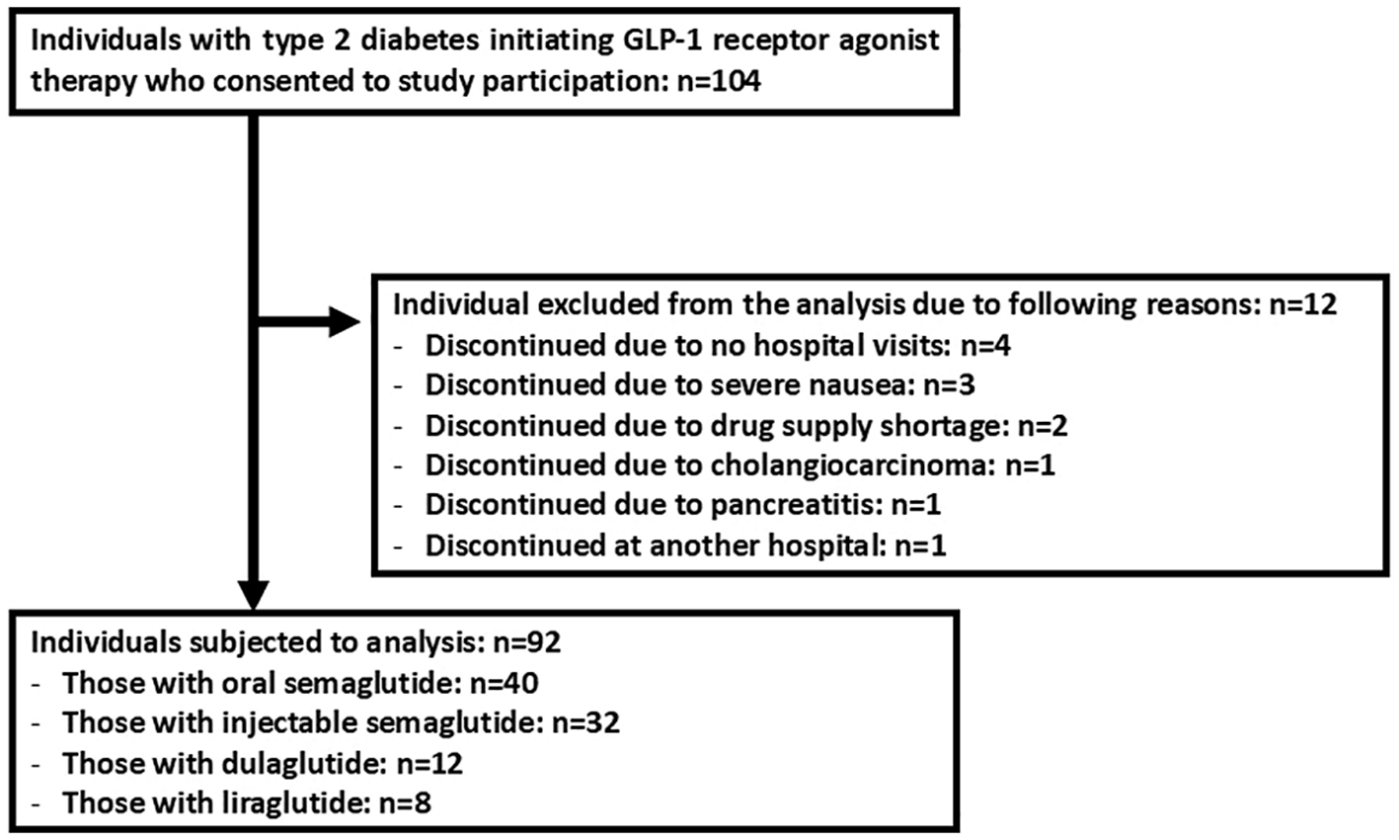
The flowchart illustrates the enrollment and reasons for exclusion from the final analysis.

A total of 104 individuals were enrolled, of whom 92 were included in the final analysis after application of the exclusion criteria (**Figure 1**). The primary reasons for exclusion were withdrawal from follow-up at the participant’s discretion, treatment discontinuation due to nausea, drug supply issues, cholangiocarcinoma, pancreatitis, and treatment cessation at a non-participating institution (**Figure 1**). The baseline characteristics of the analyzed cohort are presented in **Table 1**. The relatively consistent middle-aged profile of the study population is expected to enhance the precision of the findings. GLP-1 receptor agonists (GLP-1RAs) prescribed included oral semaglutide, injectable semaglutide, dulaglutide, and liraglutide (**Table 1**). The most commonly used concomitant anti-diabetes agents were biguanides and SGLT2 inhibitors while insulin was used in an approximately quarter of participants (**Table 1**). During the 12-month observation period, no participants initiated new anti-diabetes agents. Among the 24 participants who were receiving insulin at the time of GLP-1RA initiation, insulin doses were increased in 2 individuals. In contrast, insulin doses were reduced in 5 individuals including 1 participant who discontinued insulin entirely (**Table 2**). Among the 74 participants who were taking oral anti-diabetes agents at baseline, 1 discontinued an α-glucosidase inhibitor and 1 discontinued an SGLT2 inhibitor (**Table 2**). At 12 months post-GLP-1RA-initiation, the concomitant use of anti-diabetes agents was as follows: metformin, SGLT2 inhibitors, insulin, sulfonylureas, α-glucosidase inhibitors, glinides, and thiazolidinediones (**Table 2**). The mean daily or weekly doses of GLP-1RAs at 12 months were summarized in **Table 2**.

**Table 1:** Clinical characteristics of study participants at baseline.

	N=92
Demographic and clinical characteristics	
Age (years) (n=92)	58.1 ± 13.7
Male (%) (n=92)	58.7
Duration of diabetes (years) (n=92)	11.6 ± 11.9
Smoking history (%) (n=92)	47.8
Body weight (kg) (n=92)	80.3 ± 20.5
BMI (kg/m2) (n=92)	29.9 ± 6.7
Body fat (%) (n=86)	35.8 ± 7.9
Muscle mass (kg) (n=86)	48.2 ± 11.2
Grip strength (kg) (n=73)	29.7 ± 10.6
Selected Laboratory measurements	
Fasting plasma glucose (mg/dl) (n=70)	161.1 ± 53.4
Fasting serum CPR (ng/ml) (n=70)	2.86 ± 1.62
HbA1c (%) (n=92)	8.2 ± 1.6
AST (IU/L) (n=92)	30.0 ± 30.6
ALT (IU/L) (n=92)	36.4 ± 30.5
γ-GT (IU/L) (n=92)	51.4 ± 67.6
Total-cholesterol (mg/dL) (n=92)	190.6 ± 40.9
HDL-cholesterol (mg/dL) (n=92)	48.9 ± 12.4
Triglyceride (mg/dL) (n=92)	194.4 ± 139.3
eGFR (ml/min/1.73m2) (n=92)	70.1 ± 22.7
Dietary intake	
Total energy (kcal/day) (n=74)	1906.5 ± 589.3
Carbohydrate (kcal/day) (n=74)	316.4 ± 196.4
Protein (kcal/day) (n=74)	66.3 ± 21.5
Fat (kcal/day) (n=74)	63.3 ± 24.0
Complications and comorbidities	
Diabetic retinopathy (n, (%)) (n=92)	23 (25.0)
Diabetic neuropathy (n, (%)) (n=92)	44 (47.8)
Diabetic nephropathy (n, (%)) (n=92)	34 (37.0)
Hypertension (n, (%)) (n=92)	62 (67.4)
Hyperlipidemia (n, (%)) (n=92)	60 (65.2)
Ischemic heart disease (n, (%)) (n=92)	15 (16.3)
Stroke (n, (%)) (n=92)	10 (10.9)
Peripheral artery disease (n, (%)) (n=92)	2 (2.1)
Concomitant anti-diabetes medications	
Insulin (n, (%)) (n=92)	24 (26.1)
Sulfonylureas (n, (%)) (n=92)	4 (4.3)
Glinides (n, (%)) (n=92)	2 (2.2)
Biguanides (n, (%)) (n=92)	53 (57.6)
Thiazolidinedione (n, (%)) (n=92)	1 (1.1)
α-glycosidase inhibitors (n, (%)) (n=92)	5 (5.4)
SGLT2 inhibitors (n, (%)) (n=92)	39 (42.4)
GLP-1 receptor agonist initiated	
Oral semaglutide (n, (%)) (n=92)	40 (43.5)
Injectable semaglutide (n, (%)) (n=92)	32 (34.8)
Dulaglutide (n, (%)) (n=92)	12 (13.0)
Liraglutide (n, (%)) (n=92)	8 (8.7)

Categorical variables and continuous variables are expressed as frequency and mean ± SD, respectively. BMI, body mass index; CPR, C-peptide reactivity; HbA1c, glycated hemoglobin; AST, aspartate aminotransferase; ALT, alanine aminotransferase; γ-GTP, γ-glutamyl transpeptidase; HDL, high density lipoprotein; eGFR, estimated glomerular filtration rate; SGLT2, sodium glucose co-transporter 2; GLP-1, glucagon-like peptide-1.

**Table 2:** Changes between baseline and 12 months after GLP-1 receptor agonist initiation.

	Baseline	After 12 months	p-value
Demographic and clinical characteristics			
Body weight (kg) (n=92)	80.3 ± 20.5	76.7 ± 19.9	<0.001
BMI (kg/m2) (n=92)	29.9 ± 6.7	28.6 ± 6.2	<0.001
Body fat (%) (n=75)	35.8 ± 7.9	33.9 ± 8.8	<0.001
Muscle mass (kg) (n=75)	48.2 ± 11.2	47.8 ± 11.1	0.76
Grip strength (kg) (n=64)	29.7 ± 10.6	29.6 ± 10.6	0.83
Selected Laboratory measurements			
HbA1c (%) (n=92)	8.2 ± 1.6	7.0 ± 1.4	<0.001
AST (IU/L) (n=92)	30.0 ± 30.6	24.5 ± 13.8	0.076
ALT (IU/L) (n=92)	36.4 ± 30.5	30.4 ± 21.2	0.033
γ-GT (IU/L) (n=92)	51.4 ± 67.6	40.9 ± 52.9	0.002
Total-cholesterol (mg/dL) (n=92)	190.6 ± 40.9	179.0 ± 35.3	0.006
HDL-cholesterol (mg/dL) (n=92)	48.9 ± 12.4	51.8 ± 13.0	0.02
Triglyceride (mg/dL) (n=92)	194.4 ± 139.3	172.6 ± 115.9	0.06
eGFR (ml/min/1.73m2) (n=92)	70.1 ± 22.7	70.0 ± 21.2	0.64
Dietary intake			
Total energy (kcal/day) (n=56)	1906.5 ± 589.3	1613.7 ± 444.9	<0.001
Carbohydrate (kcal/day) (n=56)	316.4 ± 196.4	211.2 ± 60.9	0.001
Protein (kcal/day) (n=56)	66.3 ± 21.5	56.0 ± 16.8	<0.001
Fat (kcal/day) (n=56)	63.3 ± 24.0	55.4 ± 21.3	0.001
Concomitant anti-diabetes medications			
Insulin (n, (%)) (n=92)	24 (26.1)	23 (25.0)	
Sulfonylureas (n, (%)) (n=92)	4 (4.3)	4 (4.3)	
Glinides (n, (%)) (n=92)	2 (2.2)	2 (2.2)	
Biguanides (n, (%)) (n=92)	53 (57.6)	53 (57.6)	
Thiazolidinedione (n, (%)) (n=92)	1 (1.1)	1 (1.1)	
α-glycosidase inhibitors (n, (%)) (n=92)	5 (5.4)	4 (4.3)	
SGLT2 inhibitors (n, (%)) (n=92)	39 (42.4)	38 (41.3)	
GLP-1 receptor agonist initiated			
Oral semaglutide (n, (%)) (n=92)	40 (43.5)	40 (43.5) (QD 9.4 ± 4.2 mg)	
Injectable semaglutide (n, (%)) (n=92)	32 (34.8)	32 (34.8) (QW 0.7 ± 0.3 mg)	
Dulaglutide (n, (%)) (n=92)	12 (13.0)	12 (13.0) (QW 0.75 ± 0.0 mg)	
Liraglutide (n, (%)) (n=92)	8 (8.7)	8 (8.7) (QD 0.9 ± 0.1 mg)	

Categorical variables and continuous variables are expressed as frequency and mean ± SD, respectively. BMI, body mass index; CPR, C-peptide reactivity; HbA1c, glycated hemoglobin; AST, aspartate aminotransferase; ALT, alanine aminotransferase; γ-GTP, γ-glutamyl transpeptidase; HDL, high density lipoprotein; eGFR, estimated glomerular filtration rate; SGLT2, sodium glucose co-transporter 2; GLP-1, glucagon-like peptide-1.

**Figure 2:**
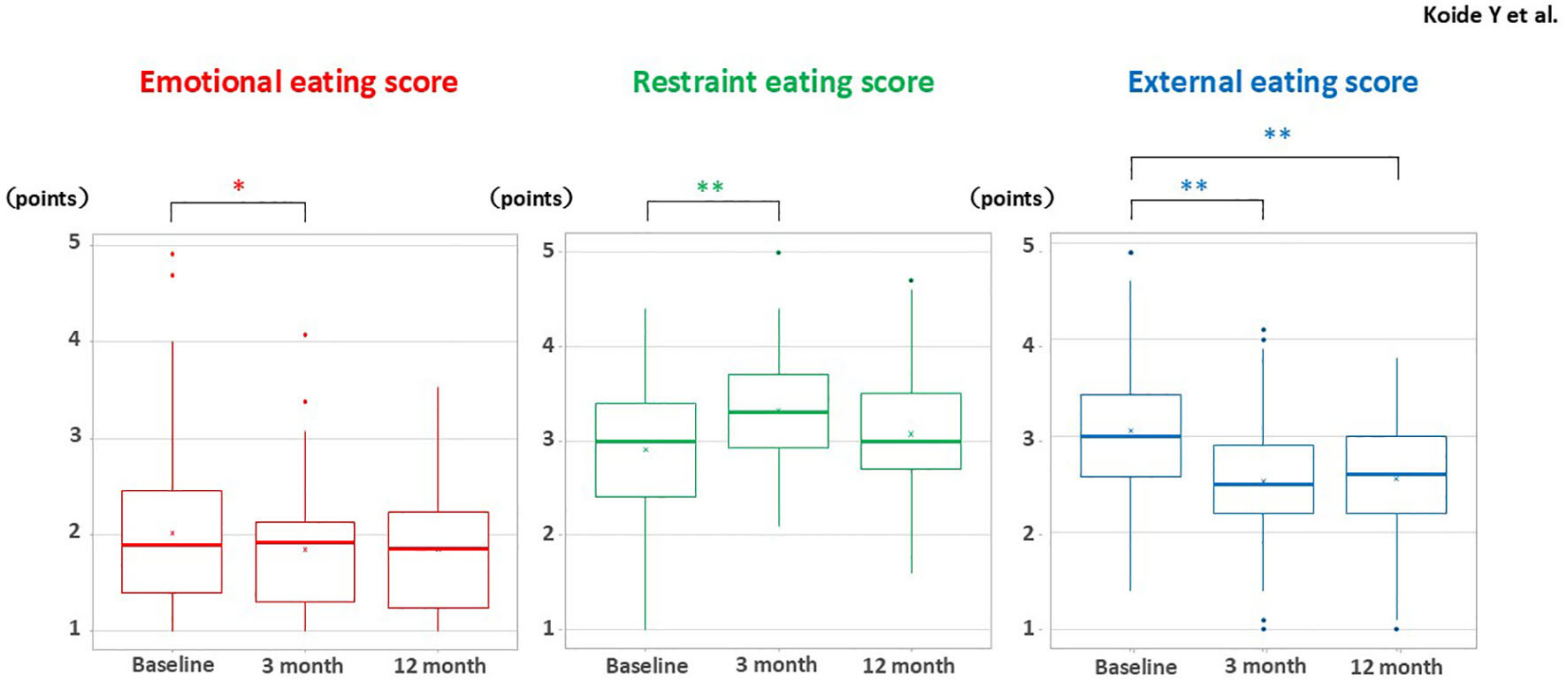
Longitudinal changes in DEBQ-J scores for emotional eating, restrained eating, and external eating at baseline, 3 months, and 12 months following initiation of GLP-1 receptor agonist therapy. Box plots display the temporal trends in eating behavior scores over the study period. Paired t-test: *p < 0.05, **p < 0.01. DEBQ-J, Dutch Eating Behavior Questionnaire – Japanese version. Twenty-two participants did not have DEBQ-J data at 12 months; therefore, the analysis was conducted with 70 participants.

At 12 months post-GLP-1RAinitiation, significant improvements were observed in HbA1c, body weight, and body fat percentage (**Table 2**). Notably, skeletal muscle mass remained unchanged despite the weight reduction. Significant reductions were also noted in ALT, γ-GTP, total cholesterol, and HDL-cholesterol levels, while triglycerides showed a trend toward improvement. Nutritional analysis via FFQ revealed significant reductions in total caloric intake and macronutrient consumption, without selective restriction of any one nutrient. Longitudinal analysis of DEBQ scores showed a significant and sustained reduction in external eating scores at both 3- and 12-months post-treatment (**Figure 2**). Emotional eating scores decreased significantly in 3 months but returned to baseline by 12 months. Restrained eating scores transiently increased in 3 months but similarly returned to baseline by 12 months. Sex-stratified analyses revealed similar patterns of changes in HbA1c, body weight, and eating behavior scores between male and female participants Multivariable linear regression models revealed that baseline HbA1c, baseline BMI, duration of diabetes, and the use of injectable semaglutide rather than liraglutide were independently associated with changes in HbA1c. However, no significant associations were found between eating behavior scores and changes in HbA1c, although a trend suggested that higher baseline external eating scores were associated with greater reductions in HbA1c (**Table 3**). In contrast, baseline HbA1c and external eating scores were independently associated with greater reductions in body weight at 12 months (**Table 4**).

**Table 3:** Multivariable linear regression analysis for changes in HbA1c 12 months after GLP-1 receptor agonist initiation.

Variable	Coefficient	95%CI	p-value
Intercept	1.86	-0.73, -4.45	0.157
GLP-1RA (Dulaglutide; Liraglutide)	0.63	0.63, -0.37	0.216
GLP-1RA (Oral semaglutide; Liraglutide)	0.79	0.79, -0.11	0.085
GLP-1RA (Injectable semaglutide; Liraglutide)	0.91	0.91, 0.04	0.041
Baseline DEBQ-J Emotional eating score	0.13	0.13, -0.21	0.446
Baseline DEBQ-J Restraint eating score	0.1	0.10, -0.26	0.576
Baseline DEBQ-J External eating score	-0.36	-0.36, -0.76	0.083
Baseline HbA1c	-0.61	-0.61, -0.76	<0.001
Baseline BMI	0.04	0.04, 0.00	0.039
Duration of diabetes	0.05	0.05, 0.03	<0.001

HbA1c, glycated hemoglobin; DEBQ-J, Dutch eating behavior questionnaire- Japanese version; CI, confidence interval, BMI, body mass index.

**Table 4:** Multivariable linear regression analysis for changes in body weight 12 months after GLP-1 receptor agonist initiation.

Variable	Coefficient	95%CI	p-value
Intercept	12.14	0.75, 23.52	0.037
GLP-1RA (Dulaglutide; Liraglutide)	-0.80	-5.46, 3.86	0.734
GLP-1RA (Oral semaglutide; Liraglutide)	-0.40	-4.52, 3.73	0.845
GLP-1RA (Injectable semaglutide; Liraglutide)	0.32	-3.71, 4.36	0.874
Baseline DEBQ-J Emotional eating score	0.42	-1.09, 1.94	0.578
Baseline DEBQ-J Restraint eating score	-0.02	-1.58, 1.54	0.981
Baseline DEBQ-J External eating score	-2.68	-4.45, -0.91	0.003
Baseline HbA1c	-0.26	-0.42, -0.10	0.002
Baseline BMI	-0.09	-0.59, 0.77	0.799
Duration of diabetes	-0.08	-0.18, 0.01	0.072

HbA1c, glycated hemoglobin; DEBQ-J, Dutch eating behavior questionnaire- Japanese version; CI, confidence interval, BMI, body mass index.

## Discussion

This multicenter, prospective observational study demonstrates that GLP-1RA therapy significantly improves glycemic control, reduces body weight, and lowers body fat percentage over a 12-month period in real-world clinical practice. Beyond these metabolic benefits, we observed that GLP-1RA therapy notably reduced external eating behaviors, a key psychological factor linked to overeating and obesity. Importantly, individuals with higher baseline external eating scores experienced greater body weight reduction, highlighting a potentially underexplored predictor of GLP-1RA efficacy.

External eating, based on externality theory, refers to the tendency to eat in response to external cues—such as the appearance, aroma, or availability of food—rather than internal physiological signals such as hunger or satiety (23, 24). This contrasts with emotional eating, which is driven by emotional arousal and negative effects (25). Although both behaviors override internal satiety cues, they differ in psychological underpinnings and clinical relevance. While some reports suggest external and emotional eating can co-occur and are both positively associated with BMI (26), external eating is less directly tied to psychological distress (27). Our findings are not necessarily aligned with prior research suggesting external eating behaviors are resistant to GLP-1RA therapy (9). The observed suppression of external eating scores at 3 months, which was sustained through 12 months, suggests that GLP-1RAs may mitigate externally driven overeating. In contrast, emotional eating scores exhibited a transient decline in 3 months but returned to baseline levels by 12 months. Furthermore, baseline emotional eating was not significantly associated with long-term changes in HbA1c or body weight, indicating a more limited therapeutic modulation of this behavior. Neuroimaging studies provide mechanistic insight into these findings. Individuals with obesity display hyperactivation of reward-related brain regions—including the insula, amygdala, orbitofrontal cortex, and striatum—when exposed to visual food cues (28). GLP-1RAs such as exenatide have been shown to attenuate this neural hyperresponsiveness (29), which could underline the reduction in external eating observed in this study. Conversely, individuals with high emotional eating scores exhibit less suppression of neural responses to food cues during GLP-1RA therapy (30), potentially limiting their responsiveness to treatment. Asking patients whether they tend to eat in response to visually appealing or tempting foods may serve as a simple and practical indicator of potential responsiveness to GLP-1RA therapy. Conversely, individuals who primarily eat in response to emotional stress may exhibit a more limited therapeutic response to GLP-1RAs. In such cases, evaluating stress-related eating behaviors could aid in selecting more appropriate pharmacological interventions. Interestingly, restrained eating did not significantly correlate with glycemic or weight-related outcomes in our cohort. Although restrained eating is typically associated with improved weight control when practiced in moderation (17), excessive dietary restraint may paradoxically contribute to disinhibited eating patterns (31). The transient increase in restrained eating observed for 3 months may reflect an initial motivational surge following GLP-1RA initiation, which dissipated by 12 months, returning scores to baseline levels.

In this study, individuals with higher baseline external eating scores experienced a significantly greater reduction in body weight, whereas the association with glycemic control showed only a non-significant trend. This discrepancy may be explained by the mechanisms through which GLP-1RAs exert their glycemic effects. The glucose-lowering action of GLP-1RAs is primarily mediated via the enhancement of insulin secretion and the suppression of glucagon secretion in a glucose-dependent manner (1), pathways that are not necessarily influenced by eating behavior patterns. However, the trend toward improved glycemic control in individuals with higher external eating scores could be partially attributed to appetite suppression induced by GLP-1RA therapy (1, 2). Reduced appetite likely leads to lower energy intake, which may indirectly contribute to improved glycemic parameters over time.

Physical activity is known to influence appetite and eating behavior. However, in this study, only standard exercise guidance, as typically provided in clinical settings, was given. Although body composition analysis revealed a reduction in body weight, skeletal muscle mass remained unchanged. The impact of GLP-1RA therapy on muscle mass has been inconsistently reported and remains incompletely understood (32). Therefore, the mechanisms underlying the preservation of skeletal muscle mass despite weight loss remain unclear and warrant further investigation.

The differences in weight-reducing effects across GLP-1RAs may be partly explained by their pharmacokinetic properties. Agents such as semaglutide and liraglutide, which have relatively low molecular weights, can cross the blood-brain barrier via GLP-1 receptors expressed on ependymal cells lining the ventricles. This enables them to act on hypothalamic and hindbrain regions involved in appetite regulation (33). In contrast, dulaglutide and albiglutide, which are fused with larger molecules (e.g., Fc fragments or albumin) to prolong plasma half-life, exhibit reduced brain penetration, which may account for their comparatively modest effects on body weight. Among different GLP-1RAs in this study, significant reductions in both HbA1c and body weight were observed with dulaglutide, oral semaglutide, and injectable semaglutide. Although liraglutide improved HbA1c, no significant weight reduction was detected, likely due to the smaller sample size within this subgroup. We did not detect significant differences between GLP-1RA agents in their impact on eating behavior patterns in this study (**Supplementary Table S1**). However, it is plausible that more pronounced effects on external eating and subsequent weight reduction could emerge in analyses restricted to lower molecular weight agents. Future investigations with larger, stratified cohorts are warranted to explore these agent-specific differences more thoroughly. Additionally, as the clinical landscape evolves, newer agents such as tirzepatide, a dual GIP/GLP-1 receptor agonist, may warrant dedicated evaluation to determine whether their unique mechanisms of action differentially influence eating behaviors and metabolic outcomes.

## Limitations

This study has several limitations that should be acknowledged. First, the observational nature of this study prevents us from establishing definitive causal relationships, and unmeasured confounders such as lifestyle factors, psychological conditions, or socioeconomic status could have influenced the results. Second, eating behaviors were assessed using self-reported DEBQ scores, which may be susceptible to recall bias or social desirability bias. Finally, our study population may have been enriched with highly motivated individuals who were more inclined to engage in lifestyle modifications and adhere to treatment, potentially limiting the applicability of our findings to the broader T2D population.

## Conclusion

In summary, this multicenter observational study provides novel insights into the interaction between GLP-1RA therapy and eating behavior patterns in individuals with T2D. Specifically, we identified external eating as a potential behavioral predictor of greater therapeutic benefit, with individuals exhibiting higher baseline external eating scores demonstrating enhanced glycemic improvement and weight loss following GLP-1RA treatment. These findings highlight the potential value of integrating behavioral assessments into personalized treatment strategies for optimizing GLP-1RA efficacy in clinical practice.

## Data Availability

The raw data supporting the conclusions of this article will be made available by the authors, without undue reservation.

## Supplementary material

Supplementary Table 1 Clinical characteristics and laboratory findings of study participants at baseline and 12 months after GLP-1 receptor agonist initiation.
