# Supplementary material for: Association between eating behavior patterns and the therapeutic efficacy of GLP-1 receptor agonists in individuals with type 2 diabetes: a multicenter prospective observational study
Source: Front Clin Diabetes Healthc. 2025 Sep 17;6:1638681. doi: 10.3389/fcdhc.2025.1638681 (PMC12483915; doi:10.3389/fcdhc.2025.1638681)
Supplement: Supplementary Table 1 — Clinical characteristics and laboratory findings of study participants at baseline and 12 months after GLP-1 receptor agonist initiation. [file Table1.docx]

**Supplementary table 1. Clinical characteristics and laboratory findings of study participants at baseline and 12 months after GLP-1 receptor agonist initiation.**

|  | Liraglutide (n=8) | | | Dulaglutide (n=12) | | | Oral Semaglutide (n=40) | | | Injectable Semaglutide (n=32) | | |
| --- | --- | --- | --- | --- | --- | --- | --- | --- | --- | --- | --- | --- |
|  | Baseline | 12-months | p-value | Baseline | 12-months | p-value | Baseline | 12-months | p-value | Baseline | 12-months | p-value |
| Age (years) | 64.3±11.3 | - |  | 69.3±10.3 |  |  | 56.1±13.7 |  |  | 54.8±12.4 |  |  |
| Male (%) | 50.0 | - |  | 10 (83.3) |  |  | 17 (42.5) |  |  | 23 (71.9) |  |  |
| Duration of diabetes (years) | 19.4±14.8 | - |  | 21.3±19.3 |  |  | 8.2±8.8 |  |  | 11.4±10.7 |  |  |
| Body weight (kg) | 75.3±9.7 | 71.1±10.6 | 0.073 | **66.9±9.6** | **64.4±9.6** | **0.033** | **81.0±25.1** | **77.7±24.3** | **<0.001** | **84.0±15.4** | **81.3±15.3** | **0.011** |
| BMI (kg/m^2^) | 30.2±4.2 | 28.5±4.9 | 0.117 | **24.9±2.2** | **23.9±2.4** | **0.033** | **31.2±8.0** | **29.6±7.6** | **0.003** | **29.9±4.5** | **29.0±4.5** | **0.010** |
| Body fat (％) | 38.5±7.5 | 35.1±7.9 | 0.117 | 32.0±6.4 | 28.8±8.9 | 0.081 | **38.3±8.3** | **36.1±8.6** | **0.024** | **34.3±7.0** | **32.6±7.7** | **0.037** |
| Muscle mass (kg) | 43.6±5.2 | 43.8±6.4 | 0.426 | 42.9±6.9 | 44.0±7.5 | 0.417 | 45.7±11.8 | 46.4±12.1 | 0.763 | 52.8±10.1 | 52.5±10.1 | 0.226 |
| HbA1c (%) | **9.1±1.3** | **7.0±1.0** | **0.020** | **9.1±1.6** | **7.3±1.0** | **0.001** | **7.7±1.5** | **6.8±1.4** | **0.001** | **7.9±1.3** | **7.1±1.5** | **0.013** |
| AST (IU/L) | 30.9±29.3 | 25.8±11.7 | 0.501 | 23.7±13.4 | 23.5±7.1 | 0.928 | 31.5±32.1 | 22.7±9.8 | 0.099 | 26.1±21.1 | 26.2±18.6 | 0.986 |
| ALT (IU/L) | 32.4±26.6 | 32.4±21.7 | 1.000 | 29.1±23.3 | 29.7±16.2 | 0.986 | **37.1±28.7** | **26.0±16.2** | **0.003** | 32.9±22.0 | 34.8±26.0 | 0.548 |
| γ-GTP (IU/L) | 66.9±52.6 | 53.3±46.6 | 0.157 | 31.1±23.3 | 27.1±13.3 | 0.323 | **46.8±41.6** | **34.5±22.1** | **0.015** | 60.6±104.7 | 51.2±81.5 | 0.175 |
| Total-cholesterol (mg/dL) | 166.5±15.6 | 169.2±20.5 | 0.618 | 171.8±29.6 | 158.3±39.6 | 0.218 | 193.3±34.9 | 185.6±36.5 | 0.053 | 198.2±50.6 | 182.0±29.6 | 0.088 |
| HDL-cholesterol (mg/dL) | 44.7±11.6 | 47.1±12.0 | 0.726 | 47.8±11.4 | 52.0±14.3 | 0.121 | 52.8±13.5 | 54.0±14.1 | 0.457 | **47.3±11.0** | **50.6±10.1** | **0.025** |
| Triglyceride (mg/dL) | 209.1±115.1 | 228.6±129.2 | 0.277 | 157.5±75.0 | 154.7±66.5 | 0.903 | 195.5±156.2 | 159.7±121.0 | 0.656 | 198.9±135.3 | 182.0±115.6 | 0.303 |
| eGFR (ml/min/1.73m2) | 59.5±25.0 | 53.6±22.1 | 0.017 | 66.3±24.1 | 66.5±18.8 | 0.581 | 75.3±23.2 | 73.1±20.8 | 0.294 | 67.2±19.3 | 69.5±19.8 | 0.444 |
| Total energy intake (kcal/day) | 1787.8±511.4 | 1544.5±96.3 | 0.471 | 1832.8±568.3 | 1461.1±389.7 | 0.227 | **1780.7±503.9** | **1571.7±465.0** | **0.002** | **2050.4±652.5** | **1713.2±446.5** | **0.010** |
| Carbohydrate intake (kcal/day) | 225.8±59.8 | 203.9±25.7 | 0.670 | 311.3±164.0 | 214.1±75.9 | 0.305 | **267.5±123.2** | **214.0±56.6** | **0.033** | **336.4±177.0** | **211.1±69.4** | **0.032** |
| Protein intake (kcal/day) | 56.4±15.3 | 51.3±11.6 | 0.560 | 61.7±13.9 | 53.4±13.6 | 0.168 | **61.9±19.6** | **54.9±18.5** | **0.008** | **73.6±23.8** | **58.7±15.0** | **0.003** |
| Fat intake (kcal/day) | 60.2±22.3 | 52.0±12.4 | 0.197 | 56.3±17.7 | 48.8±12.6 | 0.346 | **58.3±25.7** | **52.0±22.2** | **0.004** | 68.9±20.9 | 61.5±21.0 | 0.224 |
| Emotional eating score | 2.3±1.2 | 1.6±0.4 | 0.727 | 1.4±0.4 | 1.5±0.4 | 0.188 | 2.1±0.8 | 2.0±0.7 | 0.380 | 2.0±0.9 | 1.9±0.6 | 0.248 |
| Restraint eating score | 2.7±0.7 | 3.0±0.6 | 0.622 | 2.7±0.6 | 2.9±0.7 | 0.393 | 3.1±0.7 | 3.2±0.8 | 0.275 | 2.9±0.7 | 3.0±0.7 | 0.566 |
| External eating score | 3.0±0.8 | 2.6±0.5 | 0.402 | **2.8±0.9** | **2.1±0.6** | **0.014** | **3.1±0.8** | **2.5±0.6** | **0.001** | 2.9±0.6 | 2.8±0.6 | 0.331 |

Categorical variables and continuous variables are expressed as % and mean ± SD, respectively. Values showing statistical significance between baseline and 12-months after GLP-1 receptor agonist initiation are highlighted in bold. BMI, body mass index; HbA1c, glycated hemoglobin; AST, aspartate aminotransferase; ALT, alanine aminotransferase; γ-GTP, γ-glutamyl transpeptidase; HDL, high density lipoprotein; eGFR, estimated glomerular filtration rate; GLP-1, glucagon-like peptide-1.
